# Supplementary material for: Multiple measures derived from 3D photonic body scans improve predictions of fat and muscle mass in young Swiss men
Source: PLoS One. 2020 Jun 11;15(6):e0234552. doi: 10.1371/journal.pone.0234552 (PMC7289400; doi:10.1371/journal.pone.0234552)
Supplement: S1 Table — Rho RFM indicates Spearman rank correlations with RFM, and Rho SSM correlations with SMM (only Rho>0.5 are reported to provide an overview, and Rho>0.8 are reported in bold numbers). (DOCX) [file pone.0234552.s001.docx]

**S1 Table:** The 90 selected measurements, including names, system-ID, mean and standard deviation (SD). Rho RFM indicates Spearman rank correlations with RFM, and Rho SSM correlations with SMM (only Rho>0.5 are reported to provide an overview, and Rho>0.8 are reported in bold numbers).

|  | **ID-No.** | **Min** | **Max** | **Mean** | **Median** | **SD** | **Rho RFM** | **Rho SSM** |
| --- | --- | --- | --- | --- | --- | --- | --- | --- |
| **BIA** |  |  |  |  |  |  |  |  |
| Visceral fat mass (kg) | - | 0.00 | 5.33 | 0.81 | 0.53 | 0.92 |  |  |
| Relative fat mass RFM (%) | - | 0.08 | 37.82 | 14.36 | 13.07 | 7.54 |  |  |
| Absolute fat mass AFM (kg) | - | 0.05 | 48.81 | 11.45 | 9.74 | 8.26 |  |  |
| Skeletal muscle mass SMM (kg) | - | 21.28 | 41.61 | 30.64 | 30.22 | 3.80 |  |  |
| Height (m) | - | 1.64 | 1.94 | 1.78 | 1.77 | 0.07 |  |  |
| Body Mass Index BMI (kg/m2) | - | 16.41 | 37.46 | 23.26 | 22.42 | 3.65 |  |  |
| Waist circumference WC (m) | - | 0.65 | 1.16 | 0.81 | 0.79 | 0.09 |  |  |
| Weight (kg) | - | 47.45 | 129.05 | 73.83 | 71.35 | 13.58 |  |  |
|  |  |  |  |  |  |  |  |  |
| **Scanner (cm)** |  |  |  |  |  |  |  |  |
| Body height | 0010 | 164.10 | 194.00 | 177.00 | 176.25 | 6.59 |  | 0.61 |
| Head height | 0020 | 22.30 | 28.30 | 24.75 | 24.80 | 1.15 |  |  |
| Neck height | 0030 | 140.50 | 168.70 | 152.25 | 152.00 | 6.06 |  | 0.64 |
| Distance neck to buttock | 0040 | 57.00 | 67.40 | 61.62 | 61.30 | 2.32 |  | 0.53 |
| Distance neck knee | 0050 | 96.20 | 114.70 | 103.94 | 104.00 | 3.91 |  | 0.63 |
| Distance waist knee | 0060 | 58.70 | 70.90 | 63.36 | 63.10 | 2.57 |  | 0.58 |
| Distance waistband knee | 0065 | 46.70 | 63.00 | 56.07 | 56.20 | 3.04 |  |  |
| Waist height | 0080 | 103.00 | 124.80 | 111.68 | 111.60 | 4.80 |  | 0.60 |
| Buttock height | 0090 | 82.20 | 101.20 | 90.63 | 90.50 | 4.39 |  | 0.60 |
| Hip height | 0095 | 73.80 | 95.80 | 84.67 | 84.20 | 4.62 |  |  |
| Crotch height | 0100 | 69.90 | 89.80 | 78.41 | 78.40 | 4.15 |  |  |
| Knee height | 0110 | 43.80 | 53.90 | 48.30 | 48.20 | 2.35 |  | 0.60 |
| Belly circumference height | 0150 | 96.70 | 117.50 | 105.91 | 105.80 | 4.79 |  | 0.61 |
| Breast height | 0170 | 120.00 | 145.20 | 129.23 | 128.70 | 5.38 |  | 0.57 |
| Neck height front | 0180 | 132.60 | 160.80 | 144.42 | 143.85 | 6.08 |  | 0.63 |
| Upper torso torsion | 0996 | -8.10 | 6.60 | 0.00 | -0.20 | 3.12 |  |  |
| Mid neck girth | 1510 | 32.40 | 44.10 | 37.03 | 36.85 | 2.12 | 0.68 | 0.68 |
| Head circumference | 1530 | 53.80 | 61.90 | 57.68 | 57.70 | 1.57 |  |  |
| Total torso girth | 2510 | 154.40 | 193.70 | 170.72 | 169.00 | 7.83 | 0.67 | 0.79 |
| Cross shoulder over neck distance | 3010 | 36.70 | 50.40 | 41.97 | 41.40 | 2.60 | 0.59 | 0.65 |
| Shoulder angle left | 3910 | 15.30 | 37.30 | 24.81 | 24.30 | 3.96 |  |  |
| Shoulder angle right | 3911 | 17.00 | 33.50 | 25.80 | 26.15 | 3.91 |  |  |
| Width armpits | 4020 | 33.30 | 53.90 | 40.89 | 40.60 | 4.49 | 0.5 | 0.51 |
| Bust points width | 4030 | 17.60 | 25.70 | 20.56 | 20.30 | 1.73 | 0.61 | 0.73 |
| Neck front to waist distance | 4050 | 28.70 | 38.80 | 33.87 | 33.80 | 1.88 |  | 0.61 |
| Bust points around neck distance | 4070 | 59.20 | 82.10 | 67.81 | 67.30 | 4.51 | 0.68 | 0.73 |
| Bust chest girth horizontal | 4510 | 80.20 | 125.50 | 96.61 | 95.50 | 8.50 | 0.70 | 0.78 |
| Across back width armpit level | 5020 | 32.00 | 47.00 | 38.64 | 38.25 | 3.13 | 0.60 | 0.61 |
| Neck to waist center back distance | 5040 | 37.80 | 46.70 | 42.60 | 42.60 | 1.84 |  | 0.62 |
| Waist to buttock distance | 5080 | 19.00 | 26.10 | 21.99 | 21.85 | 1.16 |  |  |
| Crotch length | 6010 | 74.10 | 96.70 | 84.80 | 83.80 | 4.34 | 0.63 | 0.68 |
| Dev waist band from waist front | 6020 | -17.60 | -4.90 | -10.93 | -10.50 | 3.28 |  |  |
| Dev waist band from waist back | 6030 | -12.70 | -2.40 | -7.37 | -7.05 | 2.16 |  |  |
| Dev waist band from waist side | 6040 | -14.70 | -4.10 | -8.95 | -8.55 | 2.46 |  |  |
| Middle Hip girth | 6510 | 74.10 | 125.90 | 88.12 | 85.70 | 9.60 | **0.86** | 0.74 |
| High waist girth | 6515 | 65.40 | 116.20 | 80.27 | 78.55 | 8.92 | **0.81** | 0.70 |
| Waist band | 6520 | 70.20 | 123.10 | 85.42 | 84.00 | 9.54 | **0.84** | 0.74 |
| Waist to buttock height left | 7010 | 18.80 | 25.20 | 21.29 | 21.20 | 1.14 |  |  |
| Waistband to buttock height left | 7015 | 6.20 | 17.40 | 12.50 | 12.90 | 2.39 |  |  |
| Waist to hip thigh left | 7020 | 33.10 | 43.00 | 37.49 | 37.20 | 1.88 |  | 0.58 |
| Waist to hip thigh right | 7021 | 33.10 | 42.70 | 37.54 | 37.25 | 1.90 |  | 0.51 |
| High hip girth | 7510 | 70.70 | 127.90 | 86.53 | 84.55 | 10.69 | **0.88** | 0.72 |
| Buttock girth | 7520 | 81.20 | 130.00 | 98.30 | 97.10 | 7.60 | **0.84** | **0.83** |
| Hip girth | 7525 | 81.90 | 133.90 | 100.95 | 99.80 | 8.05 | **0.84** | **0.82** |
| Hip thigh girth | 7530 | 80.10 | 128.30 | 97.84 | 97.00 | 7.07 | 0.73 | **0.83** |
| Belly circumference | 7540 | 68.60 | 127.30 | 85.34 | 83.25 | 10.79 | **0.88** | 0.72 |
| Maximum belly circumference | 7545 | 69.80 | 127.80 | 86.07 | 84.15 | 10.76 | **0.88** | 0.72 |
| Arm length left | 8030 | 53.30 | 68.10 | 61.21 | 61.20 | 3.22 |  |  |
| Arm length right | 8031 | 54.70 | 68.80 | 61.55 | 61.85 | 3.27 |  |  |
| Upper arm length left | 8040 | 29.30 | 37.50 | 34.05 | 34.20 | 1.76 |  |  |
| Upper arm length right | 8041 | 29.40 | 38.00 | 34.14 | 34.20 | 1.81 |  |  |
| Forearm length left | 8050 | 21.40 | 30.90 | 27.16 | 27.40 | 2.02 |  |  |
| Forearm length right | 8051 | 22.10 | 33.90 | 27.40 | 27.45 | 1.92 |  |  |
| Upper arm girth left | 8520 | 23.20 | 37.40 | 29.84 | 29.30 | 2.76 | 0.71 | 0.71 |
| Upper arm girth right | 8521 | 22.80 | 36.20 | 29.94 | 29.60 | 2.89 | 0.77 | 0.69 |
| Elbow girth left | 8530 | 22.00 | 33.00 | 26.71 | 26.20 | 2.07 | 0.70 | 0.76 |
| Elbow girth right | 8531 | 22.80 | 32.50 | 26.77 | 26.50 | 2.01 | 0.64 | 0.79 |
| Forearm girth left | 8540 | 21.80 | 32.30 | 26.87 | 26.50 | 2.04 | 0.59 | **0.80** |
| Forearm girth right | 8541 | 22.70 | 32.70 | 27.09 | 26.75 | 2.03 | 0.61 | 0.79 |
| Wrist girth left | 8550 | 15.10 | 20.20 | 17.05 | 17.10 | 0.96 |  | 0.77 |
| Wrist girth right | 8551 | 14.70 | 20.30 | 16.87 | 16.80 | 0.94 |  | 0.78 |
| Upper arm diameter left | 8910 | 8.60 | 16.40 | 12.10 | 11.80 | 1.53 | 0.50 | 0.64 |
| Upper arm diameter right | 8911 | 8.90 | 17.10 | 12.45 | 12.50 | 1.59 | 0.58 | 0.66 |
| Inseam left | 9020 | 70.70 | 90.70 | 79.42 | 79.20 | 4.15 |  |  |
| Sideseam at waist left | 9035 | 103.40 | 126.20 | 112.36 | 112.25 | 4.85 |  | 0.59 |
| Thigh girth left horizontal | 9510 | 43.00 | 75.70 | 55.96 | 55.00 | 5.56 | **0.84** | 0.79 |
| Thigh girth right horizontal | 9511 | 42.50 | 76.40 | 56.47 | 55.60 | 5.75 | **0.85** | **0.80** |
| Knee girth left | 9520 | 31.30 | 49.10 | 38.05 | 37.55 | 2.78 | 0.78 | 0.73 |
| Knee girth right | 9521 | 32.60 | 48.30 | 37.90 | 37.40 | 2.73 | 0.73 | 0.76 |
| Calf girth left | 9541 | 29.30 | 47.70 | 37.09 | 36.80 | 3.41 | 0.75 | 0.74 |
| Calf girth right | 9540 | 29.20 | 47.70 | 37.07 | 36.75 | 3.32 | 0.73 | 0.75 |
| Min leg girth left | 9580 | 18.30 | 28.20 | 22.51 | 22.40 | 1.74 | 0.66 | 0.74 |
| Min leg girth right | 9581 | 18.30 | 27.40 | 22.37 | 22.25 | 1.66 | 0.66 | 0.71 |
| Overview volume (dm3) | - | 46.67 | 131.18 | 73.98 | 71.11 | 13.79 | **0.82** | **0.87** |
| Volume head (dm3) | - | 3.53 | 5.46 | 4.54 | 4.56 | 0.35 | 0.61 | 0.64 |
| Volume upperarm left (dm3) | - | 1.16 | 2.78 | 1.77 | 1.68 | 0.37 | 0.76 | 0.79 |
| Volume upperarm right (dm3) | - | 1.11 | 2.87 | 1.80 | 1.70 | 0.38 | **0.80** | **0.80** |
| Volume forearm left (dm3) | - | 0.61 | 1.62 | 1.06 | 1.04 | 0.19 | 0.51 | **0.86** |
| Volume forearm right (dm3) | - | 0.64 | 1.50 | 1.05 | 1.05 | 0.18 | 0.53 | **0.88** |
| Volume thigh left (dm3) | - | 3.87 | 11.17 | 6.95 | 6.73 | 1.41 | 0.74 | **0.85** |
| Volume thigh right (dm3) | - | 4.03 | 11.96 | 7.00 | 6.81 | 1.42 | 0.73 | **0.83** |
| Volume lower leg left (dm3) | - | 1.92 | 5.20 | 3.05 | 2.96 | 0.62 | 0.75 | **0.81** |
| Volume lower leg right (dm3) | - | 1.93 | 5.14 | 3.03 | 2.96 | 0.60 | 0.74 | **0.82** |
| Volume chest (dm3) | - | 12.41 | 33.47 | 19.50 | 18.81 | 3.77 | 0.79 | **0.81** |
| Volume belly (dm3) | - | 3.77 | 16.22 | 7.41 | 6.90 | 2.26 | 0.68 | 0.70 |
| Volume hip (dm3) | - | 8.70 | 30.65 | 13.96 | 13.22 | 3.56 | 0.72 | 0.73 |
