# Supplementary material for: Multiple measures derived from 3D photonic body scans improve predictions of fat and muscle mass in young Swiss men
Source: PLoS One. 2020 Jun 11;15(6):e0234552. doi: 10.1371/journal.pone.0234552 (PMC7289400; doi:10.1371/journal.pone.0234552)
Supplement: S1 Fig — (DOCX) [file pone.0234552.s002.docx]

**S1 Figure :** Correlation (Spearman) matrix for the three anthropometric measurements (BMI, WC, WHtR)
