# Supplementary material for: Multiple measures derived from 3D photonic body scans improve predictions of fat and muscle mass in young Swiss men
Source: PLoS One. 2020 Jun 11;15(6):e0234552. doi: 10.1371/journal.pone.0234552 (PMC7289400; doi:10.1371/journal.pone.0234552)
Supplement: Attachment — Submitted filename: Answers_R2.docx [file pone.0234552.s006.docx]

**PONE-D-19-26892 (Sager et al.): Predicting body composition from 3D laser-based photonic body scans in young men**

**Answer to the comment by Reviewer 2**

**A) Reviewer #2**

“I have the same concern with the Reviewer #1 on how the model is evaluated. And I totally agree with Reviewer#1 that the author should separate the dataset, i.e., train the model using one part and do the evaluation using the other. At lease I think the author should give that result as part of the analysis.”

- ***Answer: Thank you very much for insisting on this really important point. We agree that models should be trained and tested on different data. We would like to stress again that this is exactly what we did when we applied leave-one-out cross-validation: We trained a model on (n-1) subjects and tested it on the remaining (excluded) subject, and repeated this in turn for all subjects in the sample. This procedure avoided the arbitrary subdivision of our rather small sample into even smaller subsamples. Please let us mention that Reviewer #1, who initially suggested the dataset splitting, agreed with our explanation and with the use of this procedure for model evaluation given the aims of our study. We therefore believe that we have already sufficiently addressed this Reviewer's concern and hope that we could clarify this here.***
